# Extracellular Vesicles Released by *Leishmania* (*Leishmania*) *amazonensis* Promote Disease Progression and Induce the Production of Different Cytokines in Macrophages and B-1 Cells

**DOI:** 10.3389/fmicb.2018.03056

**Published:** 2018-12-21

**Authors:** Fernanda Marins Costa Barbosa, Talita Vieira Dupin, Mayte dos Santos Toledo, Natasha Ferraz dos Campos Reis, Kleber Ribeiro, André Cronemberger-Andrade, Jeronimo Nunes Rugani, Beatriz Helena Pizarro De Lorenzo, Ronni Rômulo Novaes e Brito, Rodrigo Pedro Soares, Ana Claudia Torrecilhas, Patricia Xander

**Affiliations:** ^1^Laboratório de Imunologia Celular e Bioquímica de Fungos e Protozoários, Departamento de Ciências Farmacêuticas, Universidade Federal de São Paulo – Campus Diadema, Diadema, Brazil; ^2^Instituto René Rachou/FIOCRUZ, Belo Horizonte, Brazil; ^3^Centro Universitário São Camilo, São Paulo, Brazil

**Keywords:** *L. amazonensis*, extracellular vesicles, macrophages, B-1 cells, cytokines

## Abstract

The extracellular vesicles (EVs) released by *Leishmania* can contribute to the establishment of infection and host immunomodulation. In this study, we characterized the shedding of EVs from *Leishmania (Leishmania) amazonensis* promastigotes. This species is the causative agent of cutaneous leishmaniasis, and its role during interactions with bone marrow-derived macrophages (BMDMs) and peritoneal B-1 cells was evaluated. *Leishmania amazonensis* promastigotes cultivated *in vitro* at different times and temperatures spontaneously released EVs. EVs were purified using size-exclusion chromatography (SEC) and quantitated by nanoparticle tracking analysis (NTA). NTA revealed that the average size of the EVs was approximately 180 nm, with concentrations ranging from 1.8 × 10^8^ to 2.4 × 10^9^ vesicles/mL. In addition, the presence of LPG and GP63 were detected in EVs obtained at different temperatures. Naïve BMDMs stimulated with EVs exhibited increased IL-10 and IL-6 expression. However, incubating B-1 cells with parasite EVs did not stimulate IL-10 expression but led to an increase in the expression of IL-6 and TNFα. After 7 weeks post-infection, animals infected with *L. amazonensis* promastigotes in the presence of parasite EVs had significant higher parasite load and a polarization to Th2 response, as compared to the group infected with the parasite alone. This work demonstrated that EVs isolated from *L. amazonensis* promastigotes were able to stimulate macrophages and B-1 cells to express different types of cytokines. Moreover, the immunomodulatory properties of EVs probably contributed to an increase in parasite burden in mice. These findings suggest that the functionality of *L. amazonensis* EVs on immune system favor of parasite survival and disease progression.

## Introduction

Extracellular vesicles (EVs) are a heterogeneous group of particles that are released by cells and play a pivotal role in intercellular communication (Théry et al., 2006). Proteins, glycoconjugates, RNA, DNA, lipids and metabolites are present in EVs and can be easily transferred from one cell to another (Campos et al., 2015; Kalra et al., 2016). In the recipient cell, EVs can exert functional effects on target molecules immediately or after fusion and the release of their contents (Torrecilhas et al., 2012). It has already been demonstrated that EVs can transfer molecules involved in drug resistance (Bouvy et al., 2017; Read et al., 2017), the regulation of cell growth (Read et al., 2017), the regulation or activation of cells of the immune system (Smallwood et al., 2016; Synowsky et al., 2017), the modulation of cellular development and differentiation (Zhou et al., 2018), and neurotransmission, among other processes (Deng et al., 2017). The diversity of functions attributed to EVs seems to be related to the great heterogeneity in their composition and biogenesis (Kowal et al., 2014; Tkach et al., 2017; Tkach et al., 2018).

In pathogens, intercellular communication by EVs can be carried out within the same species or between different species (Szempruch et al., 2016; Sampaio et al., 2018). Studies with pathogenic protozoa have demonstrated that EVs released by these parasites play an important role in their survival at different levels, such as facilitating infection in experimental models (Martin-Jaular et al., 2011; Nogueira et al., 2015), immunomodulation (Coakley et al., 2017), adaptation of the parasite to the host environment (Lambertz et al., 2012) and the transfer of resistance factors to drugs (Regev-Rudzki et al., 2013; Szempruch et al., 2016; Sampaio et al., 2018). Thus, the EVs released by some pathogens can mediate both parasite-parasite (Szempruch et al., 2016) and parasite-host (Nogueira et al., 2015; Soares et al., 2017) intercellular communication.

For *Leishmania*, previous studies demonstrated that *Leishmania donovani* promastigotes release EVs that inhibit the production of proinflammatory cytokines (such as TNF-α), promote the production of IL-10 (an immunoregulatory cytokine) by monocytes and facilitate parasite infection in C57BL/6 mice treated with parasite EVs (Silverman et al., 2010a). A similar effect was observed in BALB/c mice subjected to the co-inoculation of parasite EVs and parasites in the footpad. These animals had a significant increase in lesions, a higher parasite load and a significant increase in the expression of proinflammatory cytokines, such as IL-17 (Atayde et al., 2015). It is known that *Leishmania* species show phenotypic differences related to the differential regulation of gene expression and protein functions (Cantacessi et al., 2015). However, the mechanisms remain unknown for *L. amazonensis* EVs.

*Leishmania amazonensis* is the etiologic agent of cutaneous leishmaniasis (CL), anergic diffuse cutaneous leishmaniasis (ADCL), and disseminated cutaneous leishmaniasis (DCL) and is often naturally resistant to antileishmanial drugs (Silveira et al., 2004; Rocha et al., 2013). An interesting feature of *L. amazonensis* is its ability to promote immunological anergy by impairing the cellular immune response (Silveira et al., 2009). *L. amazonensis*, as observed for other species, infects different cell types. Macrophages are considered the main cell population since the parasites are rapidly phagocytosed and live and multiply inside these cells. To maintain survival and growth, the parasite induces a down-modulated response in macrophages and subverts the host innate defense machinery (Soong, 2012; Martinez and Petersen, 2014). B-1 cells are another cell type that is able to phagocytose the parasite (Geraldo et al., 2016). In addition, our group demonstrated that these cells are involved in parasite resistance during experimental infection with *L. amazonensis* (Gonzaga et al., 2017). However, the role of B-1 cells in *L. amazonensis* infections is less clear. Thus, as part of a wider study on *Leishmania* EVs, here we characterized their release and immunomodulatory effects on bone marrow-derived macrophages (BMDMs) and B-1 cells. In addition, we evaluated the role of *L. amazonensis* EVs in the progression of experimental leishmaniasis and their influence on the activation and/or modulation of the immune system.

## Materials and Methods

### Animals

Pathogen-free BALB/c mice (6–8 weeks of age) were purchased from the Center for the Development of Experimental Models for Medicine and Biology (CEDEME, Universidade Federal de São Paulo - UNIFESP, São Paulo, SP, Brazil). The mice were treated according to the guidelines of the National Council for Control Animal Experimentation (CONCEA) of Brazil and housed under pathogen-free conditions. All procedures were approved by the Committee on Ethics of Animal Experiments (CEUA) of UNIFESP (protocols 5070250715 and 6258130716).

### Parasites

The *L. amazonensis* strain MHOM/BR/1973/M2269 used in this project was kindly provided by Clara Lucia Barbieri (Universidade Federal de São Paulo, São Paulo, Brazil). The parasites were genotyped using restriction fragment length polymorphism (PCR-RFLP) of an *hsp70* gene fragment and the ITS1 intergenic region, as previously reported (Schönian et al., 2003; Garcia et al., 2004) (Supplementary Figure S1).

Promastigotes were cultured in 199 medium (Gibco, Life Technologies Brand, Grand Island, NY, United States) supplemented with 4.2 mM sodium bicarbonate, 4.2 mM HEPES, 1 mM adenine, 5 μg/mL hemin (bovine type I) (Sigma, St. Louis, MO, United States) and 10% fetal calf serum (FCS) (Gibco, Carlsbad, CA, United States) and maintained at 26°C until the stationary growth phase. At this stage, the parasites were recovered by centrifugation and used to obtain EVs.

### Isolation of *L. amazonensis* EVs by Ultracentrifugation

*Leishmania amazonensis* promastigotes in the stationary growth phase were recovered and washed 5 times in PBS. Approximately 10^8^ parasites were placed in each microtube and incubated in Roswell Park Memorial Institute (RPMI) medium with 2% glucose for 1, 2, 4, or 24 h at 26, 34, or 37°C for EV release (Nogueira et al., 2015). These temperatures were chosen considering the growth and evolutionary cycle of *L. amazonensis* since promastigotes are present in the vector and grow *in vitro* at 26°C, at a skin temperature of approximately 34°C and at 37°C, which corresponds to the internal temperature of the host and represents a thermal stress for the parasite in the promastigote form. Afterwards, the cultures were first centrifuged to remove parasites and debris. The viability of the parasites was then analyzed by flow cytometry (FACSCalibur) after labeling with propidium iodide (PI) (Molecular Probes, Invitrogen, Thermo Fisher, United Kingdom), according to the manufacturer’s instructions. The supernatants were filtered through 0.45-μm sterile cartridges and subjected to serial centrifugation, as follows: 500 *g* for 10 min at 4°C, 1,500 *g* for 10 min at 4°C, 10,000 *g* for 10 min at 4°C and twice at 100,000 *g* for 1 h at 4°C. The ultracentrifugation apparatus was Sorvall WX Ultra Thermo Scientific - rotor T890. The pellets were washed once and then diluted in sterile PBS.

### Microscopy and Characterization of *L. amazonensis* Extracellular Vesicles

*Leishmania amazonensis* promastigotes that were incubated for 1, 2, 4 or 24 h at 26, 34, or 37°C were fixed and subjected to scanning electron microscopy (SEM), as described previously (Soares et al., 2005; Paranaiba et al., 2017). The samples were processed and analyzed at the Center for Electronic Microscopy (CEME) at UNIFESP. For all isolated EVs from cultures of *L. amazonensis*, the protein concentrations were determined by using the Micro BCA protein assay kit (Thermo Scientific, Waltham, MA, United States). The concentration and size distribution of *L. amazonensis* EVs were measured using nanoparticle tracking analysis (NTA) in a Nanosight NS300 instrument (Malvern Instruments Ltd., Malvern, United Kingdom), equipped with a CCD camera and a 405 nm laser. Fractions were diluted 10- to 100-fold in PBS. Each sample was captured in triplicate for 30 s with the camera level set to 14. The threshold used was always the same. The data were analyzed using the NTA software (version 2.3 build 0017).

### Polyclonal Antibody Production and Size-Exclusion Chromatography

Polyclonal antibodies against *L. amazonensis* EVs were produced for use in the identification of fractions containing EVs via size-exclusion chromatography (SEC). For this purpose, the animals (*N* = 5 per group) were subcutaneously immunized with a mixture of EVs (6 μg) obtained by ultracentrifugation and complete Freund’s adjuvant (v/v) at a final volume of 0.1 mL After 15 days, all mice received a second immunization with the same number of particles emulsified in incomplete Freund’s adjuvant. The serum from the animals was collected before each immunization, and the titer was determined by enzyme-linked immunoassay (ELISA). The plates (Costar, Corning Incorporated, NY, United States) were sensitized overnight with *L. amazonensis* EVs (6 μg/mL). After blocking with BSA, serial serum dilutions were added to determine the titer after two immunizations. Peroxidase-conjugated anti-mouse IgG (Sigma, St. Louis, MO, United States) (1:2000) was added, and the reaction was performed with δ-phenylenediamine (OPD, 1 mg/mL, Sigma) in 100 mM citrate-phosphate buffer, pH 5.8, containing 0.05% (v/v) H_2_O_2_ (Perhidrol, Merck Chemicals, NJ, United States). The 4N H_2_SO_4_ stop solution was added after the color appeared. The results were evaluated by an ELISA reader (BioTek, Winooski, VT, United States) at 492 nm.

Size-exclusion chromatography was used to remove protein aggregates and to obtain high-quality EV preparations (Trocoli Torrecilhas et al., 2009; Böing et al., 2014; Nogueira et al., 2015). First, the filtered supernatants (1 mL) from cultures of *L. amazonensis* that were incubated for 4 h at 26, 34 or 37°C were concentrated in a speed vacuum (CentriVap DNA Concentrator, Labconco, Kansas City, MO, United States). Each sample was diluted in 0.1 M ammonium acetate, pH 6.5. Then, 1.5 mL of each sample was loaded onto a Sepharose CL-4B column (1 cm × 40 cm, GE Healthcare, Piscataway, NJ, United States) that was previously equilibrated with the same solution (0.1 M ammonium acetate, pH 6.5). The column was eluted with 0.1 M ammonium acetate, pH 6.5, at a flow rate of 1 mL/5 min. Then, 1 mL fractions were collected and subjected to ELISA using an anti-EV polyclonal antibody (1:200). The collected EVs were used to stimulate macrophages and B-1 cells.

### Dot Blot and ELISA

Dot-blots were performed with EVs (5 μg) or *L. amazonensis* extract (10 μg) applied to the nitrocellulose membrane. Then, membranes were blocked for 1 h with 5% milk in PBS at room temperature (RT). After blocking, membranes were probed with anti-gp63 monoclonal antibody (mAb) (1:500) for 1 h at RT. Membranes were washed three times with PBS added with 0.1% Tween 20 before their incubation with anti-mouse IgG conjugated with peroxidase (1:10,000) (KPL, SeraCare, Milford, MA, United States). After washing, the reaction was visualized using Pierce^TM^ ECL Western blotting substrate (Pierce Biotechnology, Thermo Fisher, Rockford, IL, United States).

Enzyme-linked immunosorbent assay (ELISA) was used to evaluate the presence of LPG in EVs. Plates were sensitized overnight at 4°C with *L. amazonensis* EVs (6 μg/mL) or *L. amazonensis* extract (30 μg/mL). Then, the wells were blocked with 5% milk in PBS for 1 h at 37°C. The monoclonal antibody CA7AE (1:500), that recognizes the unsubstituted Gal(β1,4)Man repeat units (Tolson et al., 1989), were added to determine the presence of LPG in EVs. After incubation for 1 h at 37°C, the plates were washed with PBS added with 0.1% Tween 20 and incubated with peroxidase-conjugated anti-mouse IgG (Sigma, St. Louis, MO, United States) (1:2000). The reaction was performed with TMB substrate solution (Pierce Biotechnology) as manufacturer’s instructions. The 2N H_2_SO_4_ stop solution was added after the color appeared and the results were evaluated by an ELISA reader (BioTek, Winooski, VT, United States) at 450 nm.

### Bone Marrow-Derived Macrophages

Bone marrow-derived macrophages (BMDMs) were obtained according to previously described procedures (Zamboni and Rabinovitch, 2003). Briefly, bone marrow cells were removed from the femurs of BALB/c mice. The collected cells were washed twice (500 g for 5 min) and cultured for 3 days at 37°C in a 5% CO_2_ atmosphere in RPMI 1640 medium supplemented with 20% heat-inactivated FCS, 100 U/mL penicillin, 100 μg/mL streptomycin (all from Sigma) and 50% conditioned medium derived from L929 cultures (LCCM) as a source of granulocyte/macrophage colony-stimulating factor. Then, the medium was removed, and the cells were maintained for 3 days under the same conditions using RPMI 1640 medium supplemented with 30% LCCM, 20% FCS, 100 U/mL penicillin and 100 μg/mL streptomycin. At the end of 4 days, the cells were cultivated using RPMI 1640 supplemented with 10% heat-inactivated FCS, 100 U/mL penicillin and 100 μg/mL streptomycin for 24 h prior to stimulation.

### Purification of Peritoneal B-1 Cells

Total peritoneal cells were obtained from a lavage of the peritoneal cavities of BALB/c mice. The total cells collected were subjected to magnetic cell sorting to obtain an enriched culture of B-1 cells by using negative selection with anti-CD23 microbeads (Miltenyi Biotec) and positive selection with anti-CD19 microbeads (Miltenyi Biotec) Gladbach, Germany) (e Brito et al., 2007; Geraldo et al., 2016). Purified B-1 cells were labeled with anti-CD11b monoclonal antibodies conjugated to phycoerythrin (PE) and anti-IgM conjugated to fluorescein isothiocyanate (FITC) (all from BD, San Diego, CA, United States) and analyzed on a FACSCalibur flow cytometer (BD) to evaluate cell purity.

### Treatment of Macrophages and B-1 Cells With *L. amazonensis* EVs

Bone marrow-derived macrophages (2 × 10^5^) and purified peritoneal B-1 cells (1 × 10^6^) were distributed in each well of a 24-well plate (Costar) and maintained at 37°C in 5% CO_2_ for 24 h. All experiments were performed with RPMI containing 10% FCS and ultracentrifugation at 100,000 *g* for 8 h at 4°C. *L. amazonensis* EVs were obtained after 4 h of parasite incubation at 26, 34, or 37°C, prepared by SEC and added to cultures of macrophages and B-1 cells. The experiments were performed with a total of 100 particles per cell, which corresponded to 2 × 10^7^ particles per well for macrophage cultures and 1 × 10^8^ particles per well for B-1 cells. The positive controls included live parasites, and the negative controls were done with the medium. The cells were incubated for 48 h at 37°C in 5% CO_2_ before cytokine expression evaluation.

### Cytokine Expression

Quantitative reverse transcriptase polymerase chain reaction (qRT-PCR) was used to evaluate the cytokine expression (Geraldo et al., 2016) in macrophages or B-1 cells treated with *L. amazonensis* EVs. All qRT-PCR procedures were developed following the MIQE guidelines (Bustin et al., 2009). Briefly, the total cellular RNA was extracted from macrophages by using the TRIzol reagent (Invitrogen); for B-1 cells, the PureLinkRNA Mini Kit (Ambion; Thermo Fisher Scientific Brand, Grand Island, NY, United^TM^ States) was used. All procedures were performed according to the manufacturer’s protocol. The total RNA was quantified by UV absorption using a spectrophotometer (Nanodrop 2000c, Thermo Fisher). Only RNA samples with reading ranges of 1.8–2.0 at 260/280 nm and 260/230 nm were subjected to electrophoresis in 1.5% agarose gels to assess RNA integrity. Next, the RNA samples that showed high quality and integrity were subjected to DNAse treatment (RQ1 RNase-free DNase; Promega, Madison, WI, United States) in identical amounts (in μg) prior to cDNA synthesis with the Proto Script First Strand cDNA Synthesis Kit (New England Biolabs Inc., MA, United States); both procedures followed the instructions of the manufacturers. The real-time PCR reactions (qRT-PCR) were performed using the SYBR Green Real-Time PCR Master Mix (Applied Biosystems, Thermo Fisher Scientific) and the StepOnePlus thermocycler (Applied Biosystems) with equal amounts of each cDNA. The sequences of the primers used for each target gene were as follows: interleukin-6 (IL-6) sense 5′-TAGTCCTTCCTACCCCAATTTCC-3′ and anti-sense 5′-TTGGTCCTTAGCCACTCCTTC-3′; interleukin-10 (IL-10) sense 5′-GCTGGACAACATACTGCTAACC-3′ and antisense 5′-ATTTCCGATAAGGCTTGGCAA-3′; tumor necrosis factor alpha (TNF) sense 5′-CCCTCACACTCAGATCATCTTCT-3′ and antisense 5′-GCTACGACGTGGGCTACAG-3′ [all sequences from PrimerBank^1^ (Spandidos et al., 2010)]; glyceraldehyde-3-phosphate dehydrogenase (gapdh) sense 5′-AAATGGT-GAAGGTCGGTGTG-3′ and antisense 5′-TGAAGGGGTCGTTGATGG-3′; and ribosomal protein, large, P0 (rplp0) sense 5′-AGCTGAAGCAAAGGAAGAGTCGGA-3′ and antisense 5′-ACTTGGTTGCTTTGGCGGGATTAG-3′. The qRT-PCR reactions were performed using the following conditions: 1 μL of cDNA, 5.0 μL of SYBR Green Master Mix (Thermo Fisher), and 2.0 μL of each oligonucleotide (1.0 μM). The cycling parameters were 10 min at 50°C for enzyme activation, denaturation for 5 min at 95°C, and 40 cycles of 95°C for 30 s and 60°C for 1 min. A non-template control was included in each reaction as a negative control to enable the detection of contamination. After evaluating the quality of the reaction based on the dissociation curves, the results obtained were analyzed for the baseline and threshold cycle values (*C*t) using the StepOnePlus software (Applied Biosystems). The baseline was always adjusted to three or two cycles prior to the detection of the fluorescent signal, and the threshold cycle (*C*t) was defined in the region of exponential amplification across all plots. All primers were previously evaluated for their efficiency by constructing standard curves using a dilution series with cDNA. The amplification efficiency was calculated using the equation *E* = 10^(-1/slope)^-1 (*E* corresponds to the efficiency, and slope is the slope of the standard curve). The relative quantification was calculated according to the 2^-ΔΔCt^ method (Schmittgen and Livak, 2008) for reactions with efficiencies ranging from 90 to 110%. The reactions were performed in triplicate using at least two biological samples. Differences in the relative expression levels of target genes were determined by comparing untreated cells as reference samples with the respective cell type stimulated with EVs or parasites. The gene expression for the reference sample was adjusted to 1.

### Measurement of Cytokines by ELISA

The levels of IL-6, IL-10, and TNF-α were measured in the supernatants of BMDM or B-1 cells treated with *L. amazonensis* EVs. The supernatants were collected, centrifuged, and stored at -70°C. All dosages were measured by capture ELISA (R&D Systems, Minneapolis, MN, United States) as described by the manufacturer. The results are expressed in pg/mL.

### Experimental *L. amazonensis* Infection

Groups of six animals were subcutaneously infected in the right footpad with 1 × 10^6^ *L. amazonensis* stationary promastigotes in the presence or absence of 1 × 10^7^ EVs obtained at 26°C. Parasites and EVs were resuspended in 50 μL of sterile, endotoxin-free PBS. Animals were checked weekly to monitor the presence of palpable edemas by monitoring the induration diameter. Footpad of animals inoculated with PBS were used as a control. To determine parasite burden, after 7 weeks the paws were aseptically removed from euthanatized mice, and individually homogenized in 199 medium. The limiting dilution method was used to determine the number of parasites (Lima et al., 1997). For histological analysis, the paws were removed, fixed in 10% neutral buffered formalin, cut into 5 μm sections, stained with hematoxylin-eosin, and examined with a Zeiss AxioScope II microscope (Carl Zeiss, Thornwood, NY, United States). Stained sections were examined to compare the intensity of the infection.

Spleen cells derived from animals infected in the presence or absence of EVs were removed and used to evaluate the cytokine production. Non-infected mice were used as negative control. Splenocytes (1 × 10^6^/well) were incubated for 5 days with EVs (25 μg/mL) obtained at 26°C. Total *L. amazonensis* extract (25 μg/mL) and culture medium were added as positive and negative controls, respectively. Supernatants were collected and used to evaluate the cytokine production.

### Cytometric Bead Array

Cytokine levels were measured in supernatants from spleen cells using a Th1/Th2/Th17 BD cytometric bead array (CBA) (BD Biosciences) following the manufacturer’s instructions. IL-2, IL-4, IL-6, IL-10, IL-17A, IFN-γ, and TNF-α were measured. Flow cytometry measurements were performed on a BD FACS Accuri C6 flow cytometer (BD Biosciences) and the data evaluated by FCAP Array^TM^ software (BD Bioscience). A total of 2,400 events were acquired for each preparation. Cytokine standards were used to construct calibration curves, which were necessary for determining the cytokine concentrations in the test samples.

### Statistical Analysis

The data are shown as the mean ± standard deviation (SD). Analysis of variance (ANOVA) followed by Tukey’s post-test was carried out to compare multiple groups. *P-*values < 0.05 were considered significant. All statistical tests were performed using Graph Pad Prism version 7 for Mac (GraphPad Software, La Jolla, CA, United States ^2^).

## Results

### Time-Dependent Release of EVs by *L. amazonensis* Promastigotes and Standardization of EV Purification by Size-Exclusion Chromatography

**FIGURE 1:**
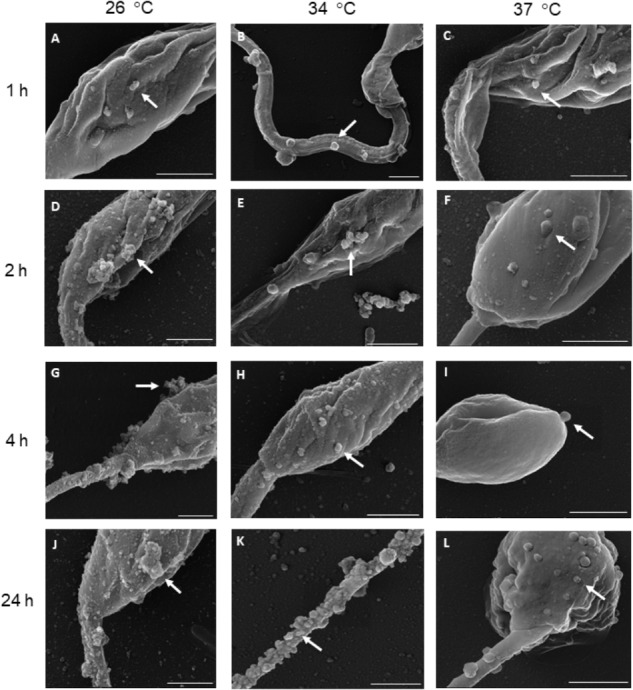
*Leishmania amazonensis* promastigotes spontaneously release EVs from their surface after incubation at 26, 34, and 37°C for **(A–C)** 1, **(D–F)** 2, **(G–I)** 4, and **(J–L)** 24 h. Parasites are seen by SEM at different magnifications. The arrows indicate EVs on the surface of the parasites. Scale bars = 1 μm.

**FIGURE 2:**
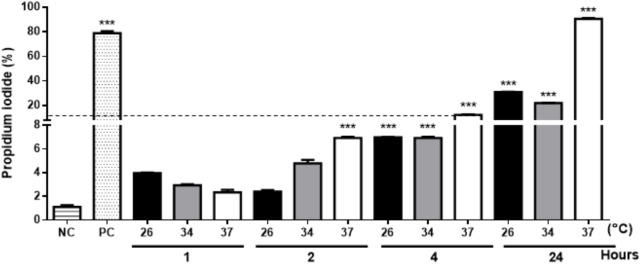
The viability of the *L. amazonensis* promastigotes was analyzed by labeling with propidium iodide (PI). Parasites cultured at 26, 34, or 37°C for 1, 2, 4, or 24 h were stained with PI and analyzed by flow cytometry. NC, negative control (fresh parasites); PC, positive control (parasites treated with 20 mM H_2_O_2_ for 1 h). The bars denote the average of three measurements, and the error bars denote the SD. ANOVA was performed, followed by a *post hoc* Tukey’s test (^∗∗∗^*P* < 0.001 compared to NC). The data are representative of two independent experiments.

Extracellular vesicles were successfully released by *L. amazonensis* promastigotes following SEM and NTA analysis. Microscopy confirmed that EV release occurred throughout the whole body at all times and temperatures analyzed (Figure 1 and Supplementary Figure S2). However, the morphology of the parasites was altered after 24 h at 34 °C (Supplementary Figure S2K) and 37°C (Supplementary Figure S2L). Due to the observed changes in the morphology of the parasites, the promastigotes were stained with PI to assess viability. Promastigotes incubated at 26, 34, and 37°C for 1 h exhibited no significant staining with PI (Figure 2). The parasites showed significant labeling after 2 h of incubation at 37°C and after 4 h at all temperatures. Nevertheless, under these conditions, the percentage of marked parasites was always below 10%. After 24 h of incubation, a higher percentage of labeled parasites was detected at all temperatures tested, suggesting an increase in parasite death.

**FIGURE 3:**
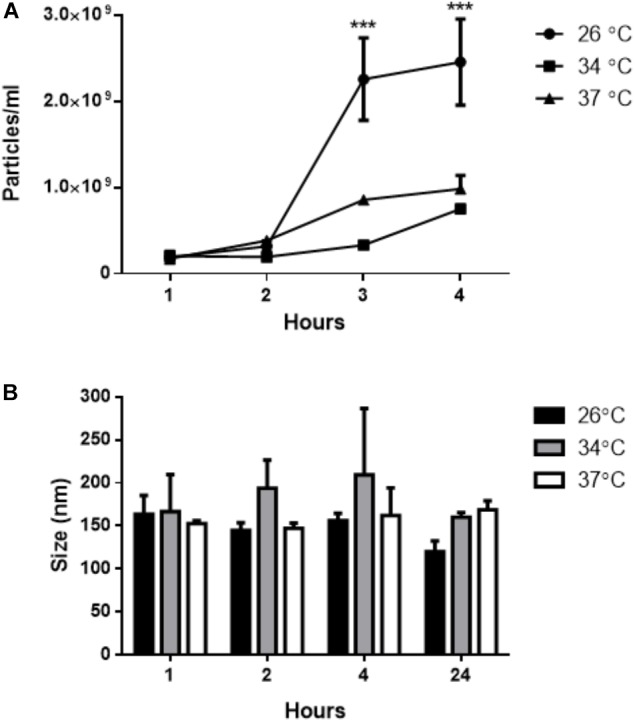
Isolation of EVs from the supernatant of *L. amazonensis* promastigote cultures by differential centrifugation. The parasites were cultured at 26, 34, or 37°C for 1, 2, 4, or 24 h. The concentration and size profile were analyzed by NTA. **(A)** The concentration (particles/mL) of EVs by NTA, and **(B)** the size profile of *L. amazonensis* EVs. ANOVA was performed, followed by a *post hoc* Tukey’s test (^∗∗∗^*P* < 0.001 compared to an initial time of 1 h). The data are representative of 3 independent experiments.

The NTA analysis revealed a time-dependent release of EVs by *L. amazonensis* promastigotes (Figure 3A) at all temperatures used. After 4 h, the number of particles was significantly higher at 26°C. The average particle size at the three different temperatures was approximately 180 nm (Figure 3B). Based on this finding, we established a time of 4 h for obtaining EVs released by *L. amazonensis* promastigotes in subsequent experiments.

**FIGURE 4:**
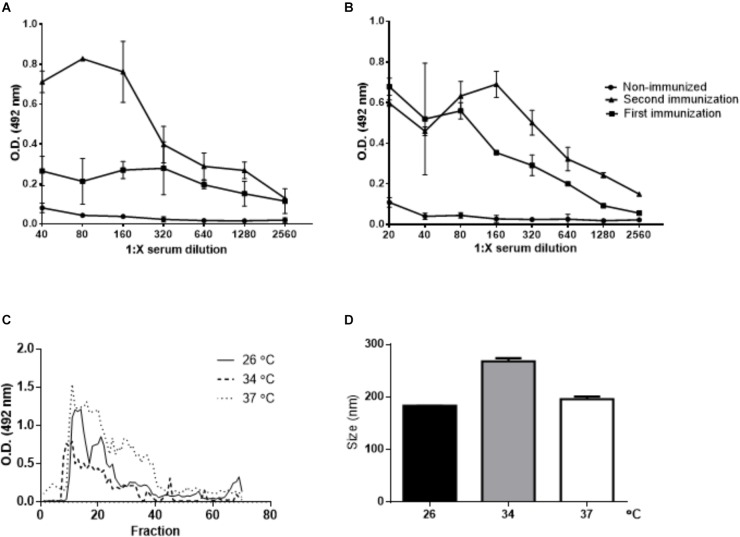
Purification of EVs released by *L. amazonensis* by SEC. Titration of the *L. amazonensis* EV-specific antibody response in BALB/c mice. Blood samples were collected before immunization (non-immunized mice), 15 days after the first immunization and 15 days after the second immunization. The level of IgG was measured in **(A)** animals immunized with EVs prepared after 4 h of parasite incubation at 26°C and in **(B)** animals immunized with EVs prepared after 4 h of parasite incubation at 37°C. Analyses were performed using ELISA with twofold serially diluted sera. The result is depicted as the mean optical density (OD) at 492 nm of five mice per group. **(C)** SEC. The promastigotes were cultured for 4 h at 26, 34, or 37°C. The presence of EVs was measured in each fraction by ELISA with anti-EV polyclonal antibodies. Seventy samples were collected, and each fraction was analyzed by ELISA. The graph shows the overlap of the measurements performed for the *L. amazonensis* EVs released at 26, 34, and 37°C. **(D)** Size profile of *L. amazonensis* EVs obtained by SEC. The particles were analyzed by NTA.

Polyclonal antibodies against *L. amazonensis* EVs were produced by immunization of BALB/c mice. After 2 immunizations, the animals exhibited a specific antibody response (Figures 4A,B). These sera were used to screen SEC fractions (Figure 4C). The positive fractions (9–30) were pooled and concentrated, and EVs were analyzed by NTA (Figure 4D).

### EVs Released by *L. amazonensis* Promastigotes Present gp63 and LPG

**FIGURE 5:**
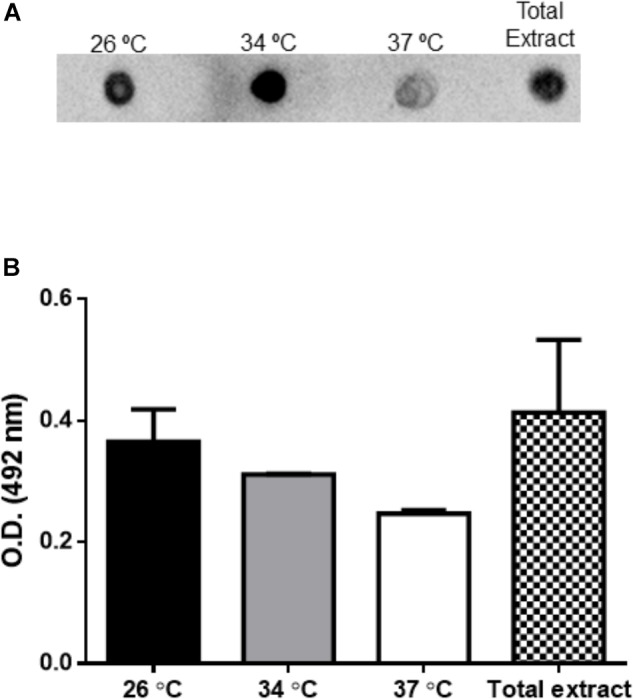
Enzyme-linked immunoassay (ELISA) and dot blotting analysis of EVs released by *L. amazonensis* promastigotes. The parasites were cultured at 26, 34, or 37°C for 4 h. The presence of gp63 and LPG were evaluated by specific mAbs. **(A)** Dot blotting of EVs (4 μg per sample) probed with the mAb anti-gp63. **(B)** The detection of LPG was performed by ELISA with mAb CA7AE.

Extracellular vesicles from *L. amazonensis* promastigotes obtained for 4 h at 26, 34, and 37°C were probed with mAb anti-gp63 by dot blot assay. The detection of gp63 is seen in EVs obtained in all temperatures analyzed (Figure 5A). Total extract from *L. amazonensis* promastigotes were used as a positive control.

Enzyme-linked immunoassay were performed with mAb CA7AE to evaluate the presence of LPG in EVs. Total extract and EVs collected from parasites incubated at 26, 34, and 37°C for 4 h were recognized by the specific mAb, suggesting the presence of LPG (Figure 5B).

### *L. amazonensis* EVs Modulate Cytokine Expression in Macrophages and B-1 Cells

**FIGURE 6:**
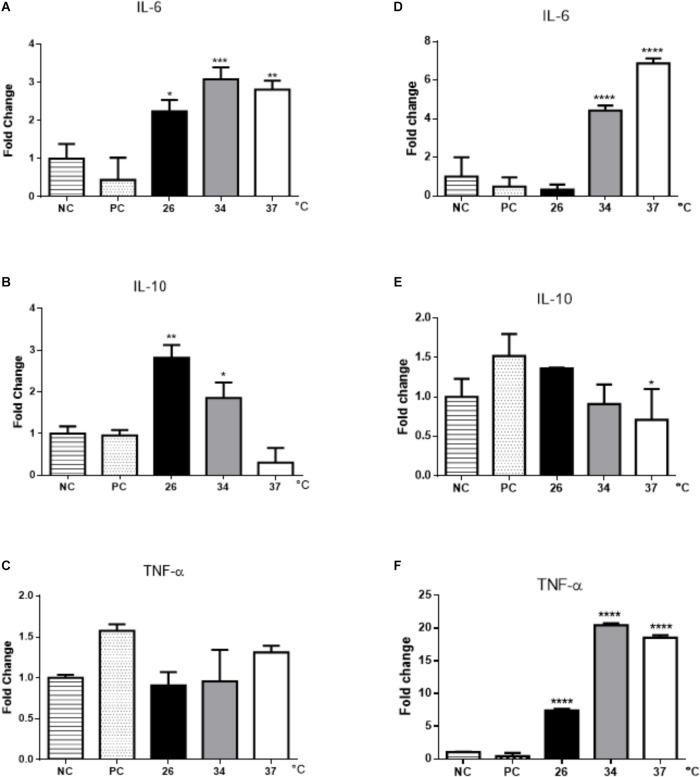
Cytokine expression in BMDMs and B-1 cells stimulated with EVs released by *L. amazonensis* promastigotes. After 48 h of stimulation with EVs, RNA was extracted, and the expression of IL-6, IL-10, and TNF- α was determined by qRT-PCR. **(A)** IL-6, **(B)** IL-10, and **(C)** TNF-α expression in BMDMs; **(D)** IL-6, **(E)** IL-10, and **(F)** TNF-α expression in B-1 cells. NC, negative control (unstimulated macrophages); PC, positive control (cells infected with live parasites). The bars show the average of three measurements, and the error bars denote the SD. The data are representative of three independent experiments. ANOVA followed by a *post hoc* Tukey’s test (^∗^*P* < 0.05, ^∗∗^*P* < 0.01, ^∗∗∗^*P* < 0.001, ^∗∗∗∗^*P* < 0.0001 compared to NC).

Cytokine expression in BMDMs was dependent on temperature. IL-6 increased in macrophages stimulated with EVs obtained at 26, 34, and 37°C, whereas IL-10 was induced only in cells stimulated with EVs from parasites incubated at 26 and 34°C (Figures 6A,B). No differences were observed in TNF-α expression (Figure 6C).

In EV-stimulated B-1 cells, an increase in the expression of IL-6 was observed, especially in cells exposed to EVs obtained at temperatures of 34 and 37°C (Figure 6D). A significant decrease in IL-10 production by B-1 cells treated with parasite EVs obtained at 37°C was observed (Figure 6E). In contrast to the results obtained for BMDMs, there was a significant increase in TNF-α expression in B-1 cells stimulated with EVs obtained at all temperatures (Figure 6).

**FIGURE 7:**
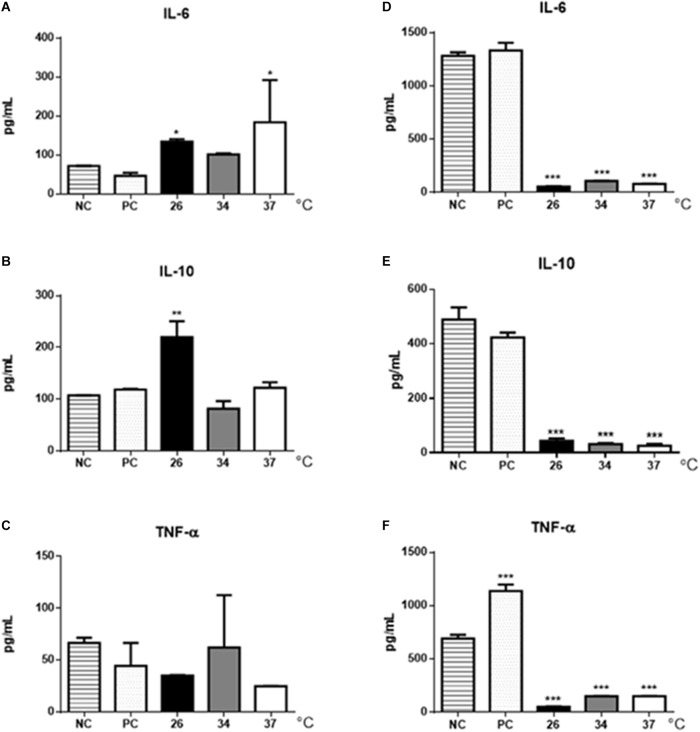
Cytokine production in BMDMs and B-1 cells stimulated with EVs shedding by *L. amazonensis* promastigotes. After 48 h of stimulation with EVs, supernatants were collected, and the production of IL-6, IL-10, and TNF- α was determined by ELISA. **(A)** IL-6, **(B)** IL-10, and **(C)** TNF-α production in BMDMs. **(D)** IL-6, **(E)** IL-10, and **(F)** TNF-α production in B-1 cells. NC, negative control (unstimulated macrophages or B-1 cells); PC, positive control (cells infected with live parasites). The bars show the average of three measurements, and the error bars denote the SD. The data are representative of two independent experiments. ANOVA followed by a *post hoc* Tukey’s test (^∗^*P* < 0.05, ^∗∗^*P* < 0.01, ^∗∗∗^*P* < 0.001, compared to NC).

The cytokine production by BMDMs and B-1 cells stimulated with EVs from *L. amazonensis* promastigotes were also analyzed by ELISA. Significant increase in IL-6 was observed in BMDMs stimulated with EVs obtained from parasites cultured at 26 and 37°C (Figure 7A) but significant increase in IL-10 production was detected only after stimulation with EVs obtained at 26°C (Figure 7B). The production of TNF-α following EVs stimulation was similar in all stimuli (Figure 7C). However, the results showed a decrease in the production of all cytokines analyzed in the culture supernatants of B-1 cells stimulated with EVs isolated from the parasites (Figures 7D–F).

### EVs From *L. amazonensis* Promastigotes Increase the Parasite Load in Mice Footpad Lesions

**FIGURE 8:**
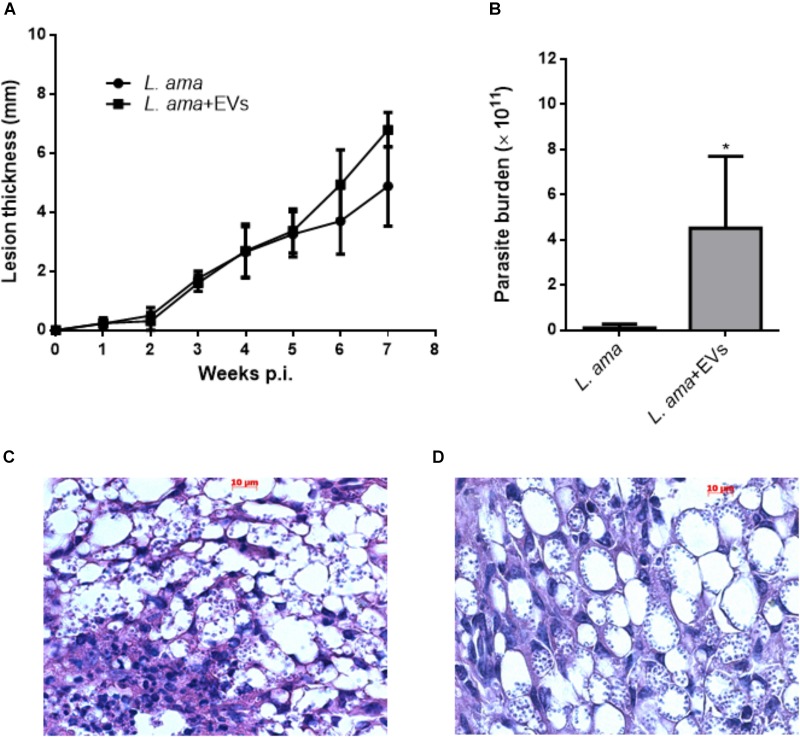
Development of infection in BALB/c mice experimentally infected with *L. amazonensis* promastigotes in the presence or absence of EVs from the parasite (*L. ama*+EVs and *L. ama*, respectively). BALB/c mice were infected in the footpad with 1 × 10^6^ *L. amazonensis* promastigotes in the presence or absence of 1 × 10^7^ EVs. **(A)** The lesion size was evaluated weekly for 7 weeks. The graph shows the measurement of the paw size and each point is representative of the mean of the measurements. **(B)** The parasite burden was evaluated by limiting dilution in the footpads (*n* = 5). Bars denote the average of 5 measurements, and error bars denote the SD. Student’s *t*-test ^∗^*P* < 0.05. **(C)** Histopathological findings in lesions after 7 weeks post-infection of BALB/c mice infected in the absence of EVs and **(D)** mice infected in the presence of EVs. The paws were removed, fixed, and 2 tissue samples from each animal were cut into 5 μm sections for staining with hematoxylin and eosin. The data are representative of 3 independent experiments.

Mice were co-injected with *L. amazonensis* stationary promastigotes and EVs obtained at 26°C. After 7 weeks post-infection, the presence of EVs in initial infection enhanced the lesion progression (Figure 8A) and significantly increased the parasite burden (Figure 8B). Histological findings also showed the lower presence of parasites in mice infected with parasites alone (Figure 8C) as compared to animals co-injected with *L. amazonensis* promastigotes and EVs (Figure 8D).

**FIGURE 9:**
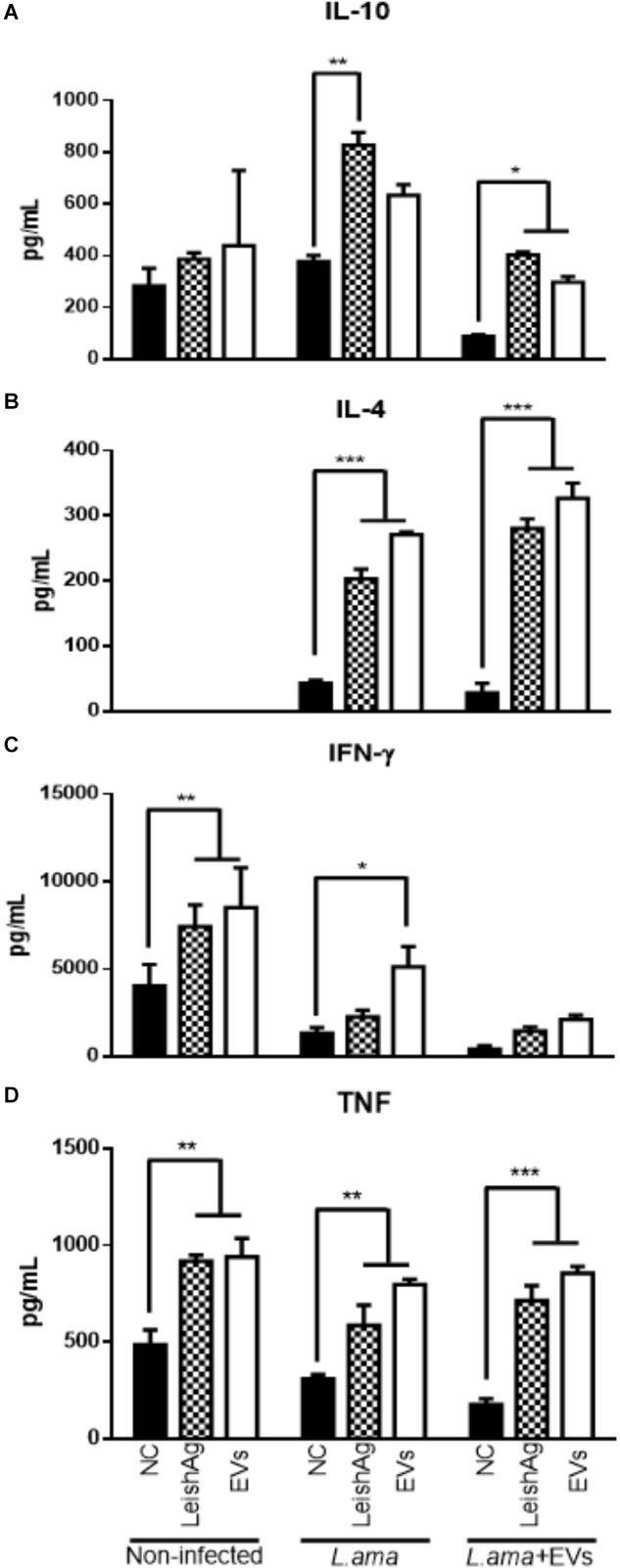
Cytokine production in splenocytes 7 weeks post-infection. BALB/c mice were infected in the footpad with 1 × 10^6^ promastigotes of *L. amazonensis* in the absence (*L. ama*) or the presence of 1 × 10^7^ EVs (*L. ama*+EVs). After 7 weeks, splenocytes were incubated with EVs (25 μg/mL) or *L. amazonensis* total extract (25 μg/mL) (positive control – LeishAg). Spleen cells from non-infected mice with and without the addition of a new stimulus were also used as controls. (NC). **(A)** IL-10, **(B)** IL-4, **(C)** IFN-γ, and **(D)** TNF-α concentrations (pg/mL) were determined by CBA. Bars express the mean value ± SD (^∗^*P* < 0.05, ^∗∗^*P* < 0.01, and ^∗∗∗^*P* < 0.001).

Cytokine production profile by splenocytes in response to *Leishmania* extract or EVs showed that animals infected with *L. amazonensis* significantly increased the production of IL-10, IL-4, IFN-γ, and TNF-α as compared to non-stimulated spleen cells (Figures 9A–D). However, splenocytes from mice infected with *L. amazonensis* in the presence of EVs had significant increase in the production of IL-10, IL-4, and TNF-α (Figures 9A,B,D) but no significant enhance in the levels of IFN-γ were observed in this group, when compared to non-stimulated cells (Figure 9C).

## Discussion

Several *Leishmania* spp. release EVs that contain virulence factors, which may interfere with the modulation of the cellular immune compartment including *Leishmania donovani* (Silverman et al., 2008, 2010b), *Leishmania mexicana* (Hassani et al., 2011), *Leishmania major* (Hassani et al., 2014), and *Leishmania infantum*/*L. major* (Atayde et al., 2015). Here, we assessed EV release by the dermotropic New World species *L. amazonensis* and the functional role of EVs during interactions with BMDMs and B-1 cells.

Similar to other *Leishmania* species, *L. amazonensis* releases EVs throughout the whole body and at different temperatures and times. However, the size of the EVs was somewhat larger (180 nm) than the sizes reported in previous studies, which ranged from 30 to 100 nm (Silverman et al., 2008, 2010a,b; Hassani et al., 2014). At 26°C, the promastigotes released larger amounts of EVs than observed at other temperatures (34 or 37°C). Increasing the temperature decreased parasite viability and morphology, thus impairing EV release. Consistent with those observations, a previous study reported that an increase in temperature affected protein secretion by *L. mexicana* (Hassani et al., 2011) and the cargo of *L. donovani* and *L. major* EVs (Silverman et al., 2010a). These data reinforce the idea that parasite adaptation to different hosts may be reflected in EV secretion and molecules. During its life cycle, *Leishmania* faces several adverse conditions, not only in the sand fly (approximately 26°C) but also in the vertebrate host (37°C) (de Assis et al., 2012). Our results showed that the production of cytokines by recipient BMDMs and B-1 cells differed depending on the temperature at which EVs were obtained.

Macrophages play a central role in the immune response to *Leishmania*. Many studies have focused on the interplay between parasites and macrophages, their phagocytic process, activation, and microbicidal mechanisms and how the parasites evade those mechanisms and grow inside these cells (Liu and Uzonna, 2012). The role of B-1 cells in leishmaniasis is not yet understood, although they are able to phagocytose *Leishmania* (Arcanjo et al., 2015; Geraldo et al., 2016) and contribute to *in vivo* resistance against experimental *L. amazonensis* infection (Gonzaga et al., 2017). Although macrophages and B-1 cells may have distinct roles in *L. amazonensis* infection, the cytokines released by these cells have pleiotropic effects. Herein, we demonstrated that EVs released by *L. amazonensis* promastigotes stimulated the expression of different cytokines by naïve BMDMs and B-1 cells.

In our work, EVs led to an increase in the expression of IL-6 and IL-10 by naïve macrophages compared to non-stimulated macrophages. The only exception was cells stimulated with EVs obtained at 37°C, which induced a decrease in the expression of IL-10. The dosage of cytokines in the BMDM supernatants showed results comparable to those observed by RT-PCR, except for the EVs obtained from parasites cultured at 34°C. Similar to our results, monocytes that were pretreated with EVs and subsequently infected with *L. donovani* showed a significant increase in IL-10 production compared to infected and non-infected macrophages (Silverman et al., 2010b). IL-10 shows anti-inflammatory effects, and its role in leishmaniasis remains controversial. High production of IL-10 has been associated with the persistence of the parasite, the establishment of chronic infection and the reactivation of the disease (Belkaid et al., 2001; Kane and Mosser, 2001; Nagase et al., 2007). In addition, IL-10 has been associated with the self-regulatory immunopathology of CL (Castellano et al., 2015). Thus, the induction of IL-10 expression in macrophages stimulated with EVs from *L. amazonensis* promastigotes could contribute to the suppression of the initial response, allowing parasite survival at the site of infection. The effects of increased IL-6 expression by macrophages treated with EVs can also be linked to parasite persistence. It has been demonstrated that IL-6 can be produced by infected resident macrophages and act on the endothelium and PBMCs to stimulate the production and release of chemokines to attract more phagocytes to the site of infection (Biswas et al., 1998; Matte and Olivier, 2002). Recently, it was demonstrated that *L. amazonensis* lipophosphoglycan (LPG), the major surface glycoconjugate, was also able to induce IL-6, TNF-α, and NO via TLR4 (Nogueira et al., 2016). The presence of LPG and GP63 in *L. amazonensis* EVs, components with immunomodulatory activity, were also identified in EVs from *L. major*, *L. infantum*, and *Leishmania enrietti* (Silverman et al., 2008, 2010b; Hassani et al., 2014; Atayde et al., 2015; Paranaiba et al., 2017). Thus, EVs from *L. amazonensis* appear to stimulate the production of IL-6 to attract more phagocytes to the site of infection and to simultaneously induce the production of IL-10 to deactivate the microbicidal effects of recruited cells. Although highly speculative, these possibilities cannot be ruled out and must await further proteomic studies.

The effects of EVs on B-1 cells were somewhat different than those observed in macrophages. B-1 cells are now considered to be an innate-like B cell population that produces natural antibodies, presents antigens, responds rapidly to the breakdown of homeostasis, and constitutively expresses IL-10 (Popi et al., 2016; Baumgarth, 2017; Savage et al., 2017). Naïve B-1 cells treated with EVs showed decreased expression and production of IL-10 compared to untreated B-1 cells. Some works have demonstrated that B-1 cell-derived IL-10 regulates immunity against some pathogens (Popi et al., 2004, 2008; Gonzaga et al., 2015; Arcanjo et al., 2017), so it was surprising that EVs released by *L. amazonensis* promastigotes induced a decrease in IL-10 expression by B-1 cells. On the other hand, although these EVs induced an increase in the expression of the proinflammatory cytokines TNF-α and IL-6, we did not detect the presence of these cytokines in the B-1 cells supernatants. In fact, B-1 cells express surface receptors for pathogen-associated molecular pattern molecules (Meyer-Bahlburg and Rawlings, 2012), and strong evidence has suggested that these cells can respond to inflammatory cues (Geherin et al., 2016). The presence of B-1 cells in an experimental model of leishmaniasis also contributes to resistance against infection by *L. amazonensis* since animals deficient in B-1 cells (XID mice) showed a higher parasite load and skin lesions with intense inflammatory infiltration (Gonzaga et al., 2017). Thus, in our study, it is possible that EVs modulated the expression of proinflammatory cytokines by B-1 cells to affect the inflammatory response to the parasite. The decreased expression of IL-10 by B-1 cells may contribute to a more efficient immune response. Taken together, these results demonstrate that EVs lead to differential cytokine expression depending on the cell type.

Our *in vivo* results showed that EVs obtained at 26°C exacerbated the disease and led to an increase in parasite load in mice infected with *L. amazonensis* promastigotes. These animals also presented splenocytes with increased IL-4 production and absence of IFN-γ after re-stimulation *in vitro*, suggesting a polarization to Th2 profile that favors the parasite growth. Our data support the model that EVs contain molecules with immunomodulatory properties (such as LPG and GP63) which may interfere with the initial modulation of innate immunity cells, such as macrophages and B-1 cells, leading to a more suppressor profile with IL-10 production. This macrophage pattern may lead to polarization to Th2, as observed in our *in vivo* experiments. In addition, the presence of IL-4 and IL-10 have been related to susceptibility in *Leishmania* infections (Sacks and Anderson, 2004). Thus, the *L. amazonensis* EVs contribute to a more permissive environment to the parasite growth which appears to be one of the main functions of the extracellular vesicles.

Some studies have reported that extracellular vesicles derived from *Leishmania* can contribute to immune evasion, pathogen survival and disease progression (Silverman et al., 2010a,b). Pre-treatment of C57BL/6 mice with exosomes from *L. donovani* increased the parasite load and generated an immune response with suppressor characteristics (Silverman et al., 2010a). Similarly, BALB/c mice showed an exacerbated disease progression and Th2 polarization after inoculation with exosomes prior to infection with *L. major* (Silverman et al., 2010b). Mice co-injected with *L. major* and exosomes derived from parasites cultured *in vitro* or derived from sand fly had a significant exacerbation of the lesions and higher mRNA expression of IL-2, IL-4, IL-17A, IL-23, IL-10, and IFN-γ in draining lymph nodes (Atayde et al., 2015). Therefore, as demonstrated to other *Leishmania* species and parasites (Trocoli Torrecilhas et al., 2009) the extracellular vesicles released by *L. amazonensis* modulate the immune response to favor the parasite growth and disease development.

Here, we showed that the effects of parasite EVs appear to depend on both the *Leishmania* species and the cell type studied. Components with immunomodulatory activity, such as GP63, were also identified in EVs from *L. major* and *L. infantum* (Silverman et al., 2008; Hassani et al., 2014; Atayde et al., 2015; Paranaiba et al., 2017). On the other hand, the quantity and composition of the molecules present in EVs may differ depending on the *Leishmania* species and the environmental conditions, and this effect can influence the type of response. In conclusion, our results support the hypothesis that *Leishmania* EVs can function as immune regulators, facilitating the establishment and survival of the parasite in the host. A better understanding of the complex interplay between *L. amazonensis* EVs and host cells, as well as the molecules (virulence factors and miRNA) involved in this cross-kingdom communication, would facilitate the elucidation of the role of *L. amazonensis* EVs in the host biological systems. In addition, understanding the escape mechanisms used by *L. amazonensis*, especially at the beginning of infection, may contribute to the identification of new molecular targets and to the development of new therapeutic approaches.

## Author Contributions

FB, MT, TD, and NR performed the experiments. KR and AT provided assistance with EV purification using size-exclusion chromatography. JR and RS helped with the genotype assays. AT assisted in SEM experiments. BL and RB assisted in the cytometric bead array. RS and AT provided several rounds of extensive feedback on both the manuscript and the figures. PX conceived and designed the study and wrote the manuscript. All authors read and approved the final manuscript.

## Conflict of Interest Statement

The authors declare that the research was conducted in the absence of any commercial or financial relationships that could be construed as a potential conflict of interest.

## Footnotes

1 https://pga.mgh.harvard.edu/primerbank/

2 www.graphpad.com
