# Supplementary figures and images for: Extracellular Vesicles Released by Leishmania (Leishmania) amazonensis Promote Disease Progression and Induce the Production of Different Cytokines in Macrophages and B-1 Cells
Source: Front Microbiol. 2018 Dec 21;9:3056. doi: 10.3389/fmicb.2018.03056 (PMC6309564; doi:10.3389/fmicb.2018.03056)

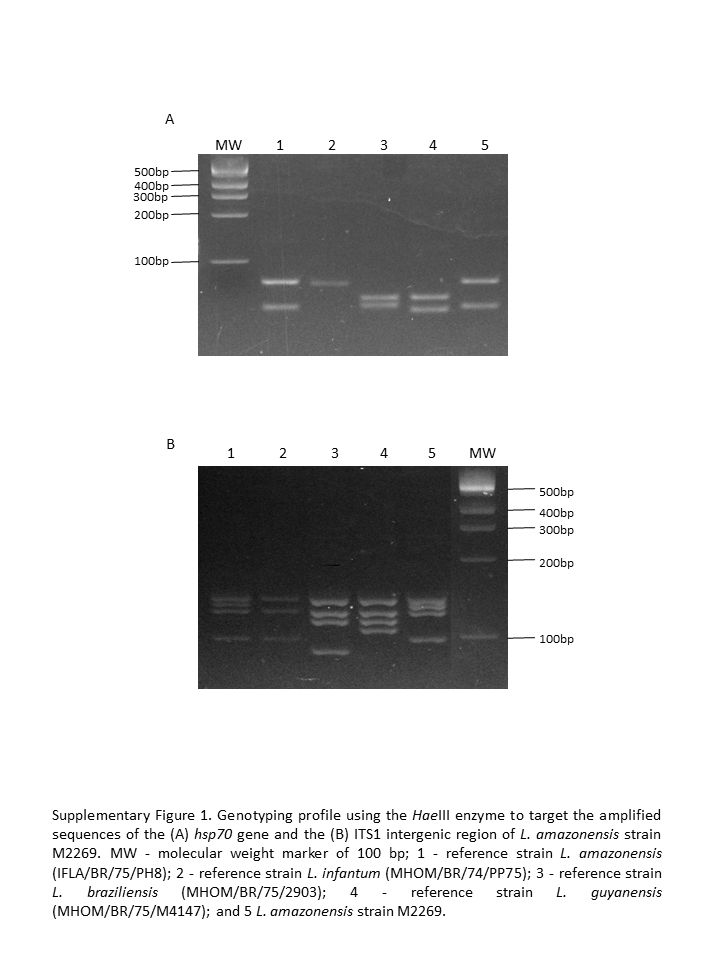

Supplement: Supplementary file 1 [file Image_1.tif]

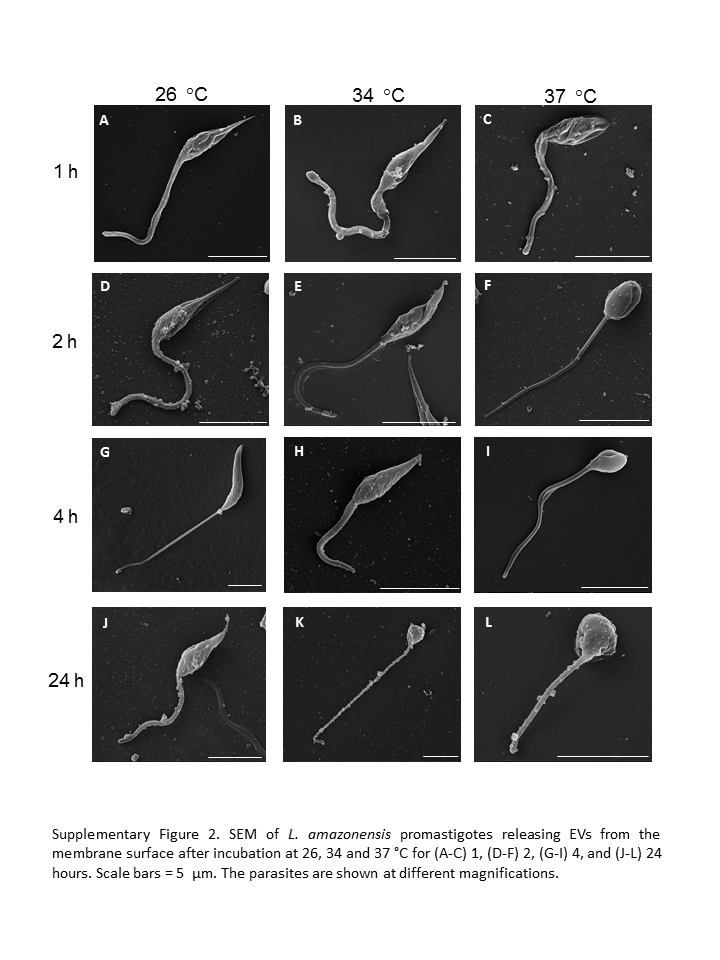

Supplement: Supplementary file 2 [file Image_2.tif]
